# Supplementary material for: Conditions during adulthood affect cohort-specific reproductive success in an Arctic-nesting goose population
Source: PeerJ. 2016 May 24;4:e2044. doi: 10.7717/peerj.2044 (PMC4888290; doi:10.7717/peerj.2044)
Supplement: Table S1 [file peerj-04-2044-s004.docx]

**Table S1.** Model-averaged estimate, 95% confidence intervals (CI) and relative importance for fixed effects in the top model set explaining variation in age at first successful reproduction among perfectly resighted Greenland white-fronted geese 1983-2003 (i.e., including only birds seen in every year of their capture history before either death or permanent emigration).

| Fixed effects | Estimate | 95% CI | Relative importance |
| --- | --- | --- | --- |
| (Intercept) | 0.00 | 0.00 | - |
| BCI^1^ | -0.23 | -0.38, -0.08 | 2.74 |
| BY M NAO^2^ | -0.03 | -0.24, 0.12 | 0.44 |
| HY M NAO*BY M NAO | 0.03 | -0.01, 0.31 | 0.45 |
| BCI*BY M NAO | -0.03 | -0.28, 0.05 | 0.43 |
| BY D NAO^3^ | 0.02 | -0.08, 0.21 | 0.38 |
| BCI*HY M NAO | -0.001 | -0.16, 0.11 | 0.07 |
| HY M NAO^4^ | -0.001 | -0.17, 0.17 | 0.02 |

^1^Breeding conditions index (BCI)

^2^Breeding year (BY) May NAO

^3^December NAO prior to successful reproduction

^4^Hatch year (HY) May NAO
